# Deep learning approach to evaluate sex differences in response to neuromodulation in Major Depressive Disorder

**DOI:** 10.1192/j.eurpsy.2022.480

**Published:** 2022-09-01

**Authors:** S. Seenivasan, M. Adamson, A. Phillips

**Affiliations:** 1 Palo Alto Veterans Affairs, Psychiatry, Palo Alto, United States of America; 2 Palo Alto Veterans Affairs, Polytrauma, Palo Alto, United States of America

**Keywords:** EEG, rTMS, deep learning, sex differences

## Abstract

**Introduction:**

Identifying the factors that mediate treatment response to rTMS in MDD patients can guide clinicians to administer more appropriate, reliable, and personalized interventions.

**Objectives:**

The present study aimed to investigate sex differences in response to repetitive transcranial magnetic stimulation (rTMS) in Major Depressive Disorder (MDD) patients.

**Methods:**

In this paper, we developed a novel pipeline based on convolutional LSTM-based deep learning (DL) to classify 25 female and 25 male subjects based on their rTMS treatment response.

**Results:**

Five different classification models were generated, namely pre/post-rTMS female (model 1), pre/post-rTMS male (model 2), pre-rTMS female responder vs. pre-rTMS female non-responders (model 3), pre-rTMS male responder vs. pre-rTMS male non-responder (model 4), and pre-rTMS responder vs. non-responder of both sexes (model 5), achieving 93.3%, 98%, 95.2%, 99.2%, and 96.6% overall test accuracy, respectively.

**Conclusions:**

These results indicate the potential of our approach to be used as a response predictor especially regarding sex-specific antidepressant effects of rTMS in MDD patients.

**Disclosure:**

No significant relationships.

